# Mammary gland adipocytes in lactation cycle, obesity and breast cancer

**DOI:** 10.1007/s11154-021-09633-5

**Published:** 2021-03-22

**Authors:** Georgia Colleluori, Jessica Perugini, Giorgio Barbatelli, Saverio Cinti

**Affiliations:** Department of Experimental and Clinical Medicine, Center of Obesity, Marche Polytechnic University, Via Tronto, 10A 60020 Ancona, Italy

**Keywords:** Mammary gland, Adipocytes, Lactation, Obesity, Breast cancer, Breastfeeding

## Abstract

The mammary gland (MG) is an exocrine gland present in female mammals responsible for the production and secretion of milk during the process of lactation. It is mainly composed by epithelial cells and adipocytes. Among the features that make the MG unique there are 1) its highly plastic properties displayed during pregnancy, lactation and involution (all steps belonging to the lactation cycle) and 2) its requirement to grow in close association with adipocytes which are absolutely necessary to ensure MG’s proper development at puberty and remodeling during the lactation cycle. Although MG adipocytes play such a critical role for the gland development, most of the studies have focused on its epithelial component only, leaving the role of the neighboring adipocytes largely unexplored. In this review we aim to describe evidences regarding MG’s adipocytes role and properties in physiologic conditions (gland development and lactation cycle), obesity and breast cancer, emphasizing the existing gaps in the literature which deserve further investigation.

## Introduction

**Fig. 1:**
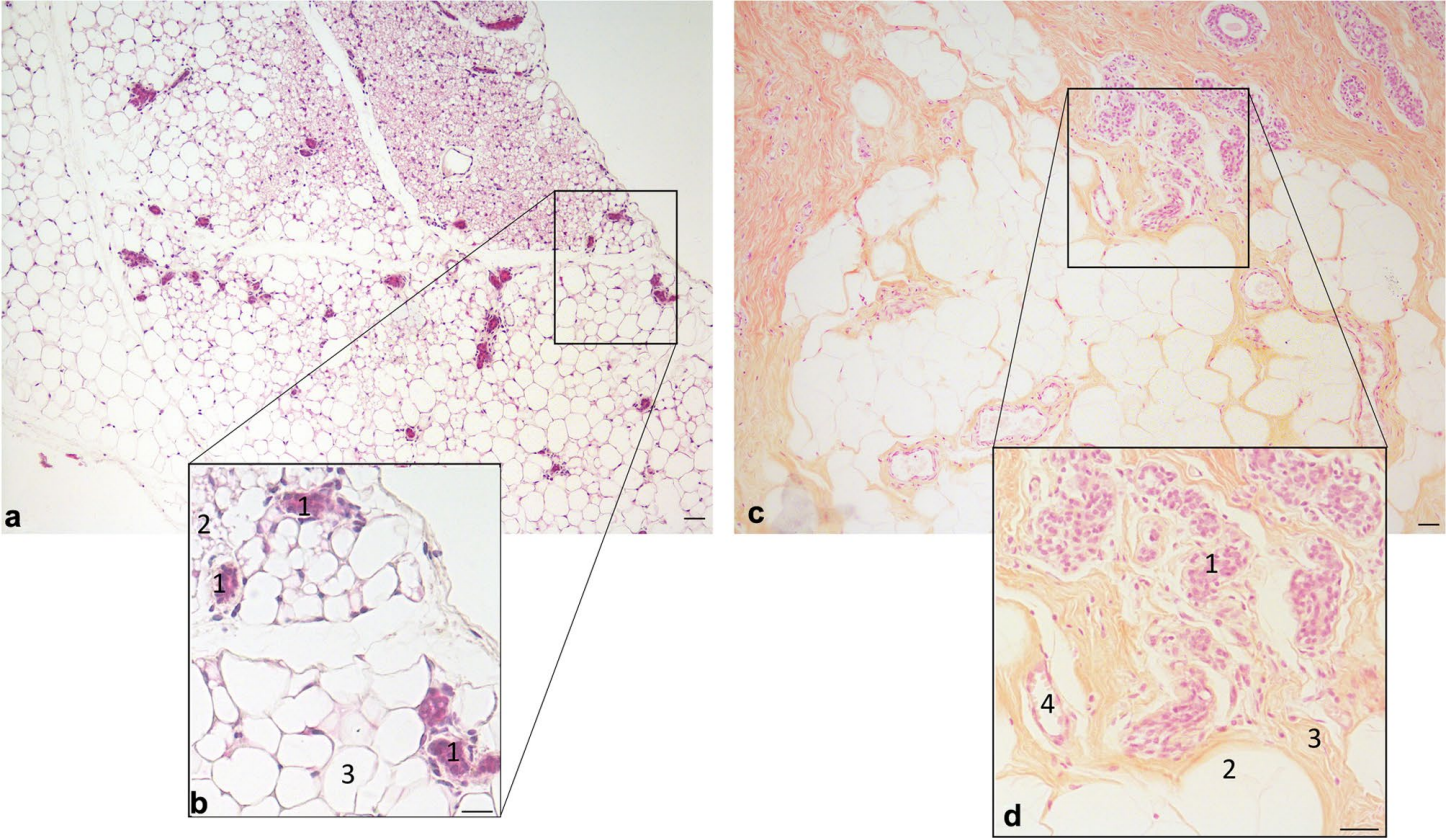
Resting Mammary Gland (light microscopy, hematoxylin and eosin staining). **a**: anterior mammary gland of a virgin C57BL/6 mouse containing white and brown adipocytes and ductal structures. **b**: magnification of squared area in A, 1: epithelial ducts; 2: brown adipocytes; 3: white adipocytes. **c**: Human mammary gland (resting): ductal structures and mammary stroma composed by white adipocytes and fibroblasts. **d**: magnification of squared area in C; 1: resting ductal structures; 2: white adipocytes; 3: fibrous stroma with fibroblasts; 4: blood vessel. Scale bars: 20μm

The mammary gland (MG) is an exocrine gland present in female mammals responsible for the production and secretion of milk during the process of lactation [1]. Lactation is the most important phenomenon able to ensure nutritional support and survival of mammal’s pups and it is considered an essential component of mammalian evolution, an evidence that further emphasize MG’s crucial role [1]. In rodents the MG is mainly composed by epithelial cells and adipocytes, while in humans the adipocytic component is relatively less represented (although still abundant) in favor of more fibrous connective tissue (Fig. 1). Among the features that make the MG unique there are 1) its high plastic properties—it in fact undergoes several cycles of remodeling consisting of a deep modification of epithelial and adipocytes morphologies during pregnancy, lactation and involution (steps belonging to the “lactation cycle”) [2]- and 2) its requirement to grow in close association with adipocytes which are absolutely necessary to ensure MG’s proper development at puberty and remodeling during the lactation cycle [3, 4]. Although MG adipocytes play such a critical role for the gland development, most of the studies have focused on its epithelial component only, leaving the role of the neighboring adipocytes largely unexplored in both, physiologic and pathologic conditions [3, 5, 6]. Only twenty-five years ago, adipocyte function has been recognized to go far beyond the mere lipid storage; the adipose tissue was in fact demonstrated to have an endocrine role regulating fundamental survival mechanisms such as eating behavior, energy homeostasis and fertility [7–9]. White adipocytes, forming white adipose tissue (WAT), store energy to supply other tissues during intervals between meals and secretes hormones capable to inform the whole body about the energy balance status [7, 10]. Brown adipocytes, forming brown adipose tissue (BAT), burn lipids for thermogenesis thanks to the unique expression of the thermogenic uncoupling protein 1 (UCP1) [7, 10–13]. WAT and BAT are organized to form a large adipose organ which occupies body’s visceral and subcutaneous compartments, including the mammary gland (MG) [7, 10]. The adipose organ is one of the players of what has been recently defined as the *nutritional system* which includes digestive organs and nuclei of the central nervous system regulating feeding behavior. These organs in fact finely cooperate with the shared objective to maintain the nutritional and energetic homeostasis [7].

The adipose depots display extremely plastic properties [10, 11]; they are capable to respond to environmental stimuli, undergoing remarkable changes in function and morphology [7, 10, 11, 14, 15]: white adipocytes can acquire a phenotype which more closely resembles brown adipocytes (known as beige adipocytes) during chronic cold exposure to increase thermogenesis, while the reverse occurs during chronic positive energy balance to expand adipose organ’s storage abilities [10, 11, 16]. Although WAT and BAT have been extensively studied due to the great clinical implication in the treatment of metabolic diseases, the MG adipocytes potentials have been weakly explored.

Further efforts are needed to deconvolute the complex and multiple functions of the adipose organ in all its different depots, including the MG. In this review we aim to describe the evidences regarding MG’s adipocytes role and properties in physiologic conditions, obesity and breast cancer, emphasizing the existing gaps in the literature which deserve further investigation.

## Mammary gland anatomy

Although beyond the scope of this review, few information concerning rodents and humans MG anatomy are reported in order to facilitate readers in the comprehension of the subsequent sections. For a deeper insight into the MG anatomy please refer to the following review articles [2, 5, 7, 17].

**Fig. 2:**
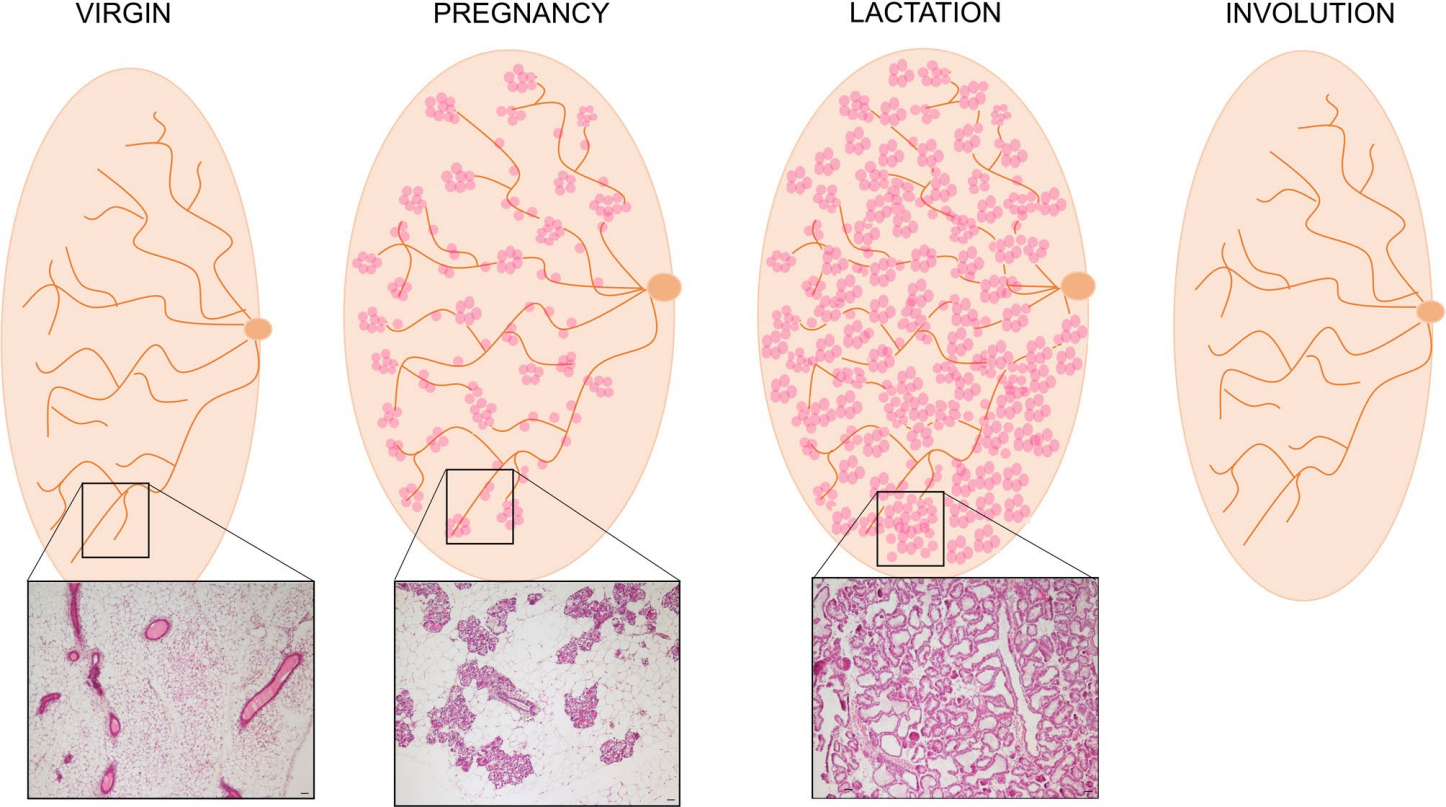
Mammary gland during the lactation cycle. Cartoon representing the mammary gland accompanied by hematoxylin and eosin staining images (mouse). Virgin: ductal structures infiltrating the mammary stroma (mainly composed by white adipocytes) converging to the nipple. Pregnancy (day 17–18): formation of alveolar structures and progressive disappearance of white adipocytes. Lactation (day 14–15): prevalence of ductal-alveolar structures and enlarged milk-producing alveoli; adipocytes are not visible. Involution: return to the pre-pregnancy mammary phenotype. Scale bars: 50μm

**Fig. 3:**
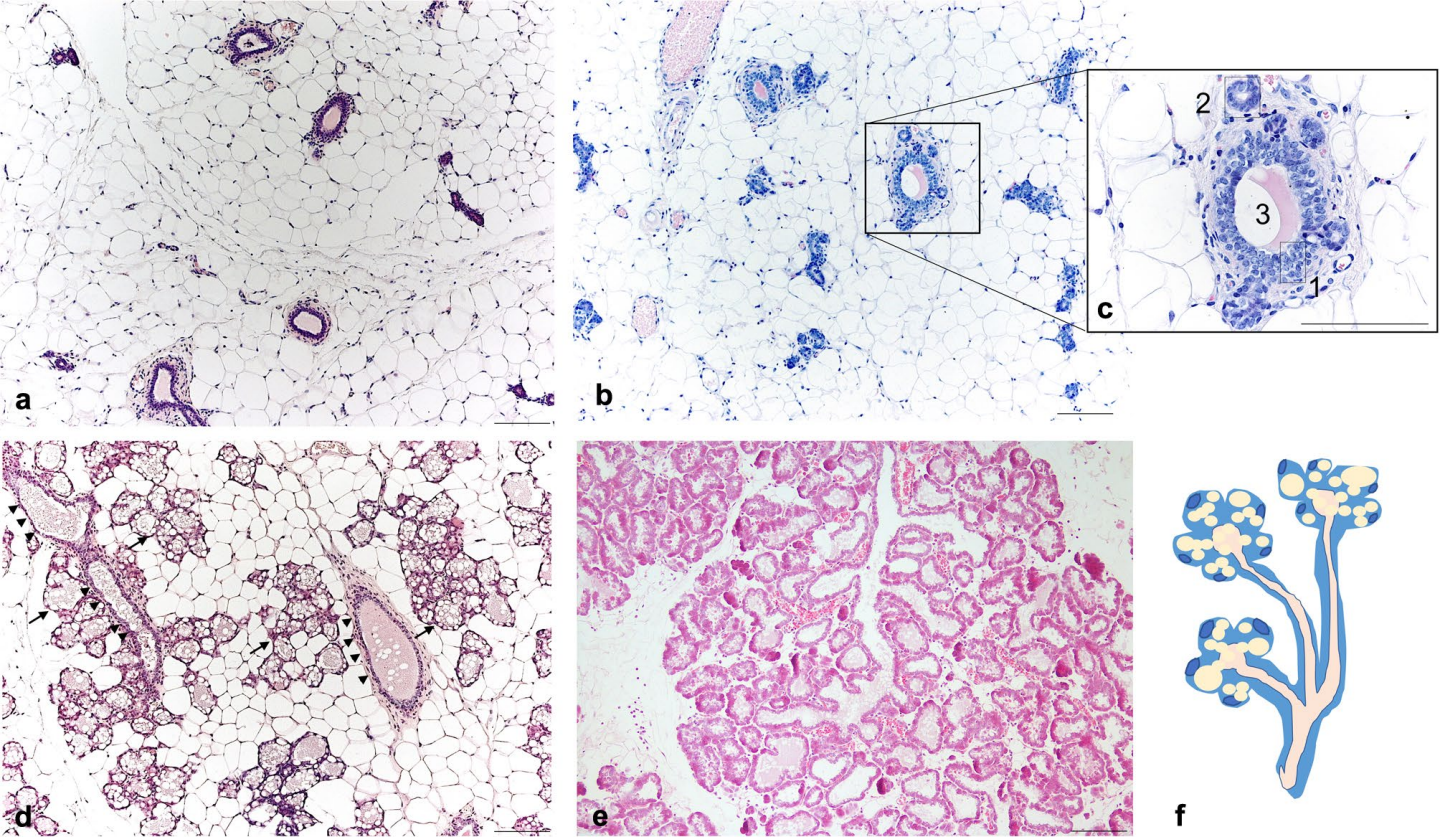
Mammary gland (mouse, 4^th^) during the lactation cycle. Light microscopy, hematoxylin and eosin staining. **a**: virgin: ductal structures infiltrating the mammary stroma mainly composed by white adipocytes. **b**: pregnancy (day 10): initial formation of alveolar structures arising from ductal stem cells. **c**: enlargement of squared area in B: 1: ductal cuboidal epithelial cells; 2: newly formed alveoli arising from ductal stem cells. 3: ductal lumen. **d**: Pregnancy (day 18): increased presence of alveolar structures containing large lipid vacuoles (arrow), ducts (arrowheads), accompanied by a progressive disappearance of white adipocytes. **e**: Lactation (day 14–15): prevalence of ductal-alveolar structures and enlarged milk-producing alveoli; adipocytes less visible. **f**: Cartoon representing a ductal-alveolar structure. Scale bars: 50μm

In rodents there are five pairs of mammary glands, three located in the cervico-toracic area, anteriorly, and two in the inguinal posterior area [2]. Anteriorly, the gland lays in continuity with the interscapular dorsal adipose depot, reason for which brown adipocytes are also part of this structure (Fig. 1a) [18]. Posteriorly it infiltrates the subcutaneous adipose depot (composed by three parts dorsolumbar, inguinal and gluteal), mainly composed by white adipocytes [2]. It is worth mentioning that most of the research studies are performed on the fourth mammary gland (inguinal) [3] and that what is usually indicated as “mammary fat pad” is also composed by endothelial (blood vessels), immune cells and fibroblasts, although adipocytes are the main cell type of the mammary stroma (Fig. 1b) [5]. In humans, there are two MG composed of glandular and adipose tissues supported by a loose framework of fibrous connective stroma (Fig. 1c, d). The glandular component is formed by lobes containing mainly ductal structures, last of which converge into one nipple. In both species, the main macroscopic MG components consists of lactiferous ducts infiltrating the subcutaneous mammary fat pad (Fig. 1 and 2). The ductal structures are composed by epithelial cuboidal luminal cells (inner layer) surrounded by an outer layer of basal, myoepithelial cells in contact with the basal membrane (see Fig. 3c). Interposed within luminal and myoepithelial cells there are mammary stem and progenitor cells which consist of different subpopulations able to give rise to the basal and luminal lineages. The complexity of the mammary epithelial hierarchy has been recently emphasized by different *single cell RNAseq* studies which however report conflicting data and whose discussion goes beyond the scope of this review [5, 6, 19, 20]. Morphofunctional modification of the MG during pregnancy will be discussed in Sect. 4.

## Adipocytes role during mammary gland development

Unlike other organs, the MG completes its development at puberty and reaches a mature functional state only at pregnancy [2, 17]. In rodents, the MG formation starts at embryonic day (E) 10.5, when the ectoderm gives rise to the mammary line, and continues up to E18.5 [5]. At E12-E13, placodes originated from the mammary line become buds of epithelial cells, and form a distinct structure from the surrounding epidermis. MG adipocytes starts differentiating at around E16-17 and allow the proper epithelial localization within the fat pad [21]. While the mammary epithelium remains quiescent from birth to puberty, MG adipocytes develop into their typical phenotype three days after birth [5]. MG ultrastructural studies evidenced that adipocytes arise from perivascular mesenchymal cells and progress from a state of preadipocytes, with few small lipid vacuoles and well-defined organelles, to the one of mature white adipocytes with one big unilocular lipid vacuole occupying most of the cytoplasm [22].

At puberty the epithelial ductal components infiltrate and occupy the whole subcutaneous adipose tissue forming several branched structures which merge in one of the nipples [2] (Fig. 2, virgin). Infiltration and elongation occur thanks to the intense mitotic activity of terminal end bud structures, which lay at the end of each lactiferous ducts and guide the formation of the ductal tree at puberty. Such infiltration at puberty is dependent on the action of estrogens since ovariectomized and estrogen receptor deficient mice display defective MG development [23, 24]. Importantly, estradiol also favors hyperplasia and hypertrophy of MG adipocytes, with new adipocytes developing in closed proximity of ductal structures [22]. Consistently, the MG of ovariectomized mice lacking estrogens are rich in multilocular adipocytes in closed proximity of blood vessels and inguinal lymph node [22]. MG growth impairments are also observed in lipodystrophic models (LD) (lacking adipose tissue) which display a sterile phenotype rescued by leptin injections [25]. In particular, the MG in these mice are underdeveloped with the lack of terminal endbuds and display a phenotype that is not rescued by either estradiol administration or by normalizing adipokines levels. Nevertheless, when the MG epithelium from LD mice is isolated and transplanted into wild type (WT) animals containing normal adipose tissue, the gland develops properly. This finding emphasizes the need of a direct cell to cell interaction between epithelial cells and adipocytes for the proper MG development which cannot rely on the mere presence of functional, but distal, adipose tissue [25]. Additional intriguingly data come from the study of ob/ob mice characterized by having a loss of function mutation in the leptin gene. The lack of leptin results in an impairment in the function of the hypothalamic-pituitary gonadal axis, thus in the abnormal estradiol secretion. This model in fact, besides suffering from obesity, diabetes and hepatic steatosis, are also not fertile and do not completely develop the MG. The phenotype of this model can be rescued by leptin treatment [25, 27]. Collectively, these data suggest that all, estradiol, leptin and adipocytes-epithelial cell to cell interaction are required (but individually not sufficient) for the proper MG development and functioning [25]. Adiponectin, an adipokine exclusively produced by adipocytes, has been identified among the mediators of the mentioned cell to cell interaction [28]. Mice models with adiponectin deficiency or overexpression display impaired alveolo-epithelial development during pregnancy and lactation [28]. Importantly, it is possible that further additional molecules mediate the MG-cell to cell interaction reason for which additional studies exploring such aspect are required.

It is worth mentioning that the functional role of brown adipocytes during MG development has not been characterized. According to some evidences, BAT negatively regulates MG epithelial differentiation during prepubertal ductal growth [18]. Selective depletion of MG brown adipocytes (UCP1-DTA model) in fact, resulted in an increased number of TEBs and ductal branching, with an evident epithelial differentiation into premature alveoli expressing the milk protein *β-casein* at 5 weeks of age [18]. It is thus possible that brown and white MG adipocytes play different functions during the gland development which still need to be thoroughly evaluated.

## Mammary gland adipocytes role during the lactation cycle

During the lactation cycle, which includes pregnancy, lactation and gland involution at weaning, the MG undergoes remarkable morpho-functional changes in its adipocytic and epithelial components. Specifically, at pregnancy adipocytes progressively “disappear” leaving space to newly formed milk producing alveoli, which enlarge at lactation. Contrariwise, during gland involution (at weaning) adipocytes become again visible and the MG acquires back a phenotype similar to the one observed in virgins (Fig. 2 and 3) [2]. Such phenomenon emphasizes the extreme plasticity of MG adipocytes, making it an interesting field of investigation. However, what happens to adipocytes during such events is not entirely clear and the evidences describing adipo-epithelial remodeling dynamics are somewhat conflicting.

The main adipocytes’ recognized role is to store lipids in the form of triglycerides to supply energy to other tissues in condition of needs (e.g. starvation). For what concern the MG adipocytes, the most accepted theory is that they release lipids to alveoli during pregnancy, thus contributing to milk production, and then get filled back with the left-over lipids from alveoli during gland involution. According to such model, adipocytes would get “slimmer” at pregnancy and enlarge in the post-lactation period thanks to the lipid trafficking from a cell type to another. Experimental evidences in favor to this last step have been reported by Zwick and colleagues in 2018 who studied the mechanisms behind adipocytes expansion during involution [4]. Specifically, the group demonstrated that “slimmed” adipocytes acquire lipids from alveoli during the first days of involution and that their expansion is not explained by the proliferation of adipocytes precursors [4]. The experiments also emphasized the fundamental role of adipocytes in orchestrating lipid trafficking and involution: the selective depletion of adipocytes using Adiponectin^Cre^R26^mT/mG^; inducible Diphtheria Toxin Receptor (iDTR) mice (model used for temporal, site specific, and inducible deletion of mammary adipocytes via diphtheria toxin administration) hampered epithelial regression and led to alveoli lipids’ retention at weaning [4]. In this context, it is important to point out the evidences described by Brenot and colleagues who studied LD mice and reported that milk production does not depend on the local presence of functional adipocytes in the MG, but instead on a functional hormonal milieu determined by healthy adipose depot (even far from the gland) [25]. In support of adipocytes’ crucial role during the lactation cycle, there are studies compromising adipocytes function through selective knock-out or knock-down approaches. For example, adipocytes selective removal of the central regulator of endoplasmic reticulum adaptive responses XBP1, led to increased adiposity and compromised lactation [29], while the removal of adipocyte’s *Pxmp2* (regulator of peroxisome) hampered MG development at pregnancy [30]. Consistent findings emphasizing the crucial role of functional adipocytes during the lactation cycle have been also collected from diet induced obesity models [31] and will be discussed in Sect. 6.

**Fig. 4:**
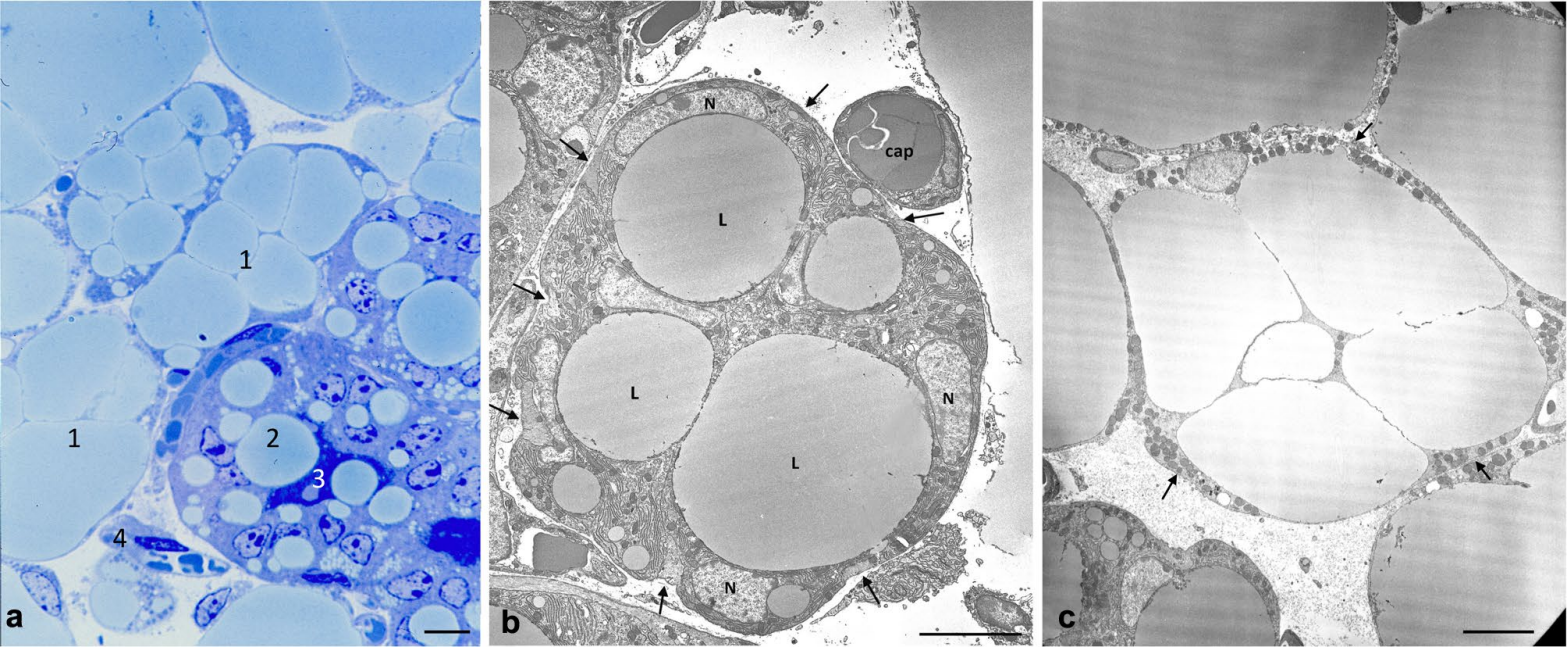
Mammary gland at day 17–18 of pregnancy. **a**: light microscopy image of a resin embedded and toluidine stained tissue: 1: compartimentalized adipocytes; 2: lipid rich alveoli; 3: alveolar lumen; 4: blood vessel. **b**: Transmission electron microscopy of one representative early alveolus (before lumen formation). L: lipid droplet; N: nucleus of epithelial cells; cap: capillary; arrows: myoepithelial cells. **c**: compartimentalized adipocyte by TEM; arrows indicate an unusually enlarged cytoplasm containing numerous organelles. Scale bars: 5μm

**Fig. 5:**
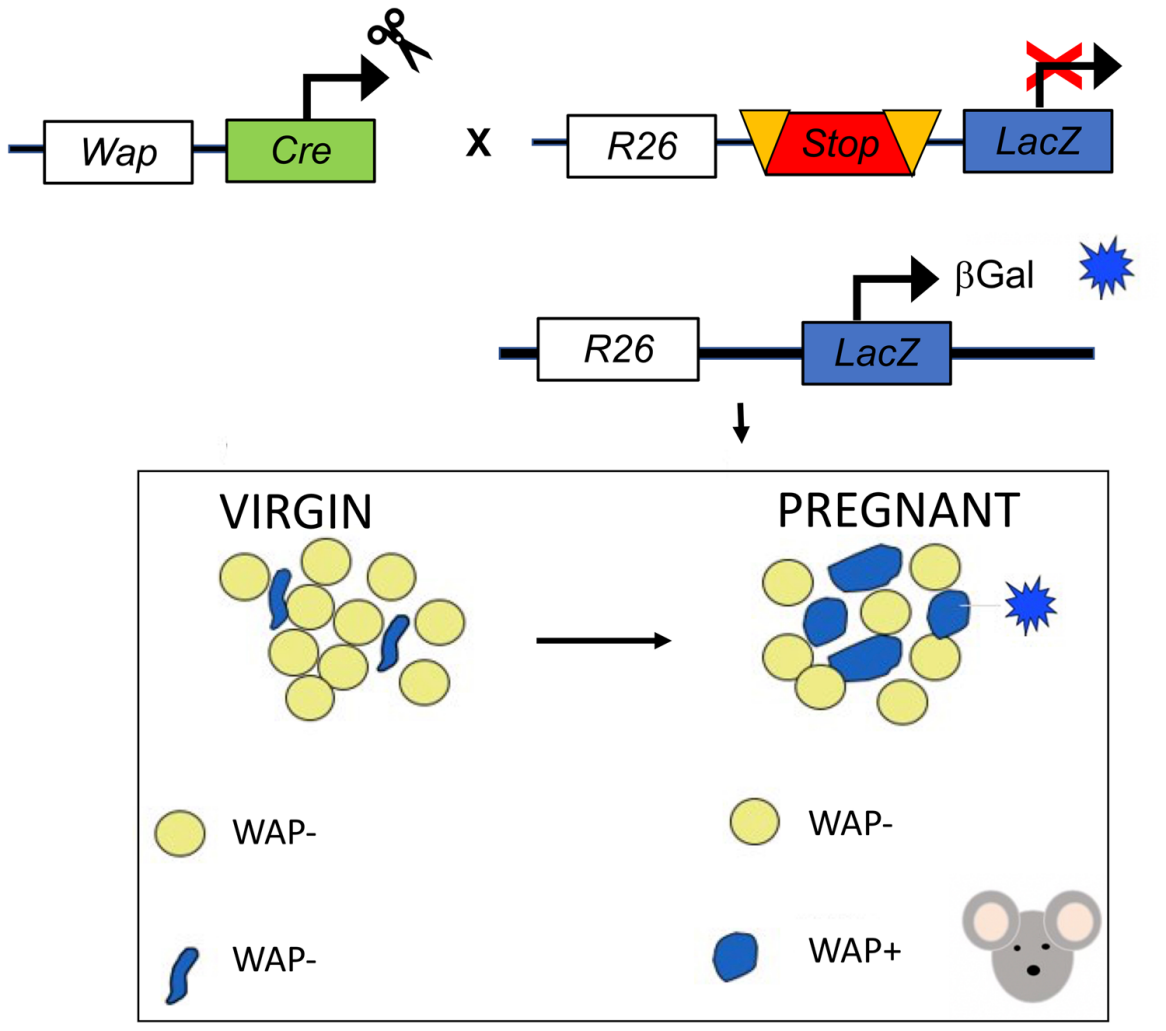
Schematic illustration of a lineage tracing experiment; WAP^Cre^R26^LacZ^ mice. In this model, most of the milk-producing cells expressing the *Whey Aciding Protein,* WAP, undergoes recombination (mediated by the Cre recombinase) and start constitutively expressing the reporter gene (LacZ) which allow to effectively visualize such cells by β-Gal staining reactions. Virgin mice do not display any positively stained cell as compared to pregnant mice

Going back to the remodeling dynamics, Zwick and colleagues were also able to isolate “slimmed” adipocytes during lactation and demonstrated that they maintain a gene expression profile typical of mature adipocytes [4]. Such evidence is partially in contrast with another study published during the same year by Wang and colleagues who demonstrated that adipocytes do not merely lose their lipids but dedifferentiate into PDGFRα+ preadipocytes and fibroblast like cells during pregnancy and keep such phenotype throughout lactation [32]. Afterwards, during gland involution, these cells proliferate and differentiate back into mature adipocytes, a phenomenon that, according to the authors, occurs during multiple pregnancies. These data revealed that mature adipocytes, widely recognized as post-mitotic cells, can undergo dedifferentiation, proliferation and then can differentiate back in their mature phenotype multiple times in physiologic conditions. On the other hand, another elegantly performed study on the MG demonstrated that PDGFRα+mesenchymal preadipocytes give rise to epithelial cells during pubertal mammary growth, and to lobuloalveolar structures at pregnancy [33]. Specifically, such phenomenon would occur in condition of stimulation with a hormonal milieu that mirrors the one typical of the progesterone dominant phase at pregnancy. Interestingly, the responsible mechanism consists of sex hormones-induced PDGF signaling in epithelial cells, which mobilize PDGFRα+mesenchymal progenitors, eliciting their migration in a chemotactic manner [33]. Considering these last two described studies, one could wonder whether mature adipocytes are capable to dedifferentiate into PDGRFα+precursors and then differentiate into lobuloalveolar cells at pregnancy. Published and unpublished data from our group support MG adipocytes (white) capability to transdifferentiate into milk secreting epithelial cells during pregnancy and return to their adipocytic phenotype at involution (both white and brown) [26, 34–36]. Specifically, during late pregnancy (mice at 17-18^th^ day) we noticed cells with an intermediate phenotype between adipocytes and alveolar structures (Fig. 4) which stained positively for both, alveologenesis and mature adipocytes markers [35, 36]. Furthermore, transplants of marked mature adipocytes into the mammary gland of a virgin mouse gave rise to stained alveoli during pregnancy [36]. These evidences were also supported by lineage tracing experiments [34–36]. Such kind of experimental approach is used to mark permanently a specific cell type in order to track its destiny throughout its entire lifespan. For example, in order to mark all milk producing cells, a WAP^Cre^R26^LacZ^ model was employed by our group [34, 35]. In this model, most of cells expressing the *Whey Aciding Protein,* WAP (milk-producing cells), underwent recombination (induced by the cre recombinase) and started constitutively expressing the reporter gene (LacZ) which allows to effectively visualize such cells by staining reactions. A schematic representation of a lineage tracing experimental model is reported in Fig. 5. In our experiments using such model, we observed not only blue alveoli, which was expected due to their ability to produce milk at lactation, but also blue stained adipocytes in the post-lactation period. These data were further confirmed by the presence of β−Galactosidase derived crystals in this post-lactation adipocytes by electron microscopy, an evidence that made the immunohistochemistry results unequivocal [35]. Such evidence led us to believe that, at weaning, a large part of the adipocytes arise from epithelial cells [34, 35]. A consistent finding, supporting adipoepithelial transdifferentiation at pregnancy, was revealed using the Ap2^Cre^R26^LacZ^ model, which at the time of the experiment was known to specifically track mature adipocytes destiny [35]. However, the Ap2 gene was found to be expressed also by other cell types and evidences from later studies using Adiponectin^Cre^ models were not able to confirm such phenomenon, questioning the existence of the transdifferentiation [4, 32, 33]. Nonetheless, the group of Speakman demonstrated that up to 2,5% of the myoepithelial cells of the antero-dorsal gland arise from the transformation of UCP1 + brown and beige adipocytes, supporting the transdifferentiation theory [37]. According to our proposed model, alveolar cells would arise from both ductal stem cells (Fig. 3) and in part also from mature adipocytes transformation, even though we cannot exclude that an intermediate step of dedifferentiation, as opposed to the direct transdifferentiation, may occur.

Based on this conflicting data, it is evident that the exact biological events characterizing MG adipo-epithelial remodeling during the lactation cycle still need to be elucidated [3]*.* Considering the inconsistent data obtained through lineage tracing experiments (WAP^Cre^ vs Adiponectin^Cre^), it would be useful to study such phenomenon at a single cell level in order to discern the different cellular populations and differentiation status during the lactation cycle. Another experimental approach able to specifically study mature adipocytes gene expression profile during the lactation cycle, could be the use of Adiponectin^Cre^R26^NuTrap^ mice, a model that overcomes the technical limitations faced during mature adipocyte cells sorting. In this model, mature adipocytes’ ribosomes can be selectively immunoprecipitated to isolate ribosome-bound mRNA and study adipocytes expression signature by *RNAseq* or RT-qPCR [38].

Similarly to what described in the MG development section, the specific role of brown adipocytes in the context of the lactation cycle has not been functionally characterized. Thermogenesis is suppressed during lactation [39] and the overexpression of *Pgc1α* (which establishes the brown adipocyte phenotype), results in precocious MG involution at pregnancy [40]. Our group demonstrated that mammary epithelial cells transdifferentiate into brown adipocytes in the post-lactational period [34], reason that made us believe that the reversible transdifferentiation between brown adipocytes and epithelial cells secreting milk may occur during the lactation cycle [2]. However, such phenomenon requires further investigations and still needs to be explored in depth.

## Mammary gland adipocytes secretory function

Since its discovery, the endocrine function of the adipose tissue has opened new groundbreaking lines of research. Proteomic studies revealed the secretion of over 350 adipokines by the MG adipose depot [41]. Adipokines secreted into the circulation act as hormones, being able to target distant organs regulating their function. In the MG, adipocytes-secreted molecules also exert a paracrine role regulating MG development and remodeling during the lactation cycle. However, the investigation of the effect of local adipokines as opposed to the circulating ones on MG development, remodeling and on milk production are lacking. Importantly, different evidences underlined the presence of adipokines into the milk, suggesting they may also exert a regulatory function in neonates. In this section we briefly describe evidences investigating the role of adipocytes secreted molecules within the MG.

MG adipocytes produce several adipokines and growth factors such as leptin, adiponectin, insulin growth factor 1 (IGF-1), vascular and endothelial growth factor (VEGF) and hepatocyte growth factor (HGF). Leptin importance in the development and remodeling of the MG has been emphasized in studies of genetic models lacking leptin (ob/ob), its receptor (db/db) or from LD mice whose phenotype, characterized by sterility and lack of MG development, is rescued by leptin injection [25, 28, 42]. However, such features are related to leptin’s systemic effect and studies isolating the role of leptin secretion within the MG fat pad are scarce. Leptin regulates milk production in synergy with prolactin, also produced by mature adipocytes, and mediates the expression of ERα in mammary epithelial cells [21, 42]. Its presence in the milk is higher during the first week postpartum (colostrum), suggesting that a certain amount is produced within the MG, since its circulating levels remain constant [42]. Importantly, milk leptin content was reported to be greater in obese models [43]. Similarly to leptin, the content of adiponectin in the milk was reported to be associated with adiposity by different studies, although the correlation between maternal BMI and milk concentration of these adipokines is still under debate [44]. Differently from leptin, adiponectin is negatively regulated by prolactin, and is less expressed in the lactating MG [21]. Mice lacking adiponectin are characterized by epithelial developmental defects at pregnancy and lactation [45]. Specifically, they have a lower number of alveolar structures and display early involution (day 10 of lactation). On the other side, adiponectin overexpression leads to a lower epithelial expansion during pregnancy and lactation, resulting in the presence of a persistently elevated number of adipocytes [45]. Consistently, in wild-type animals, adiponectin expression levels parallel MG adipocytes presence during the lactation cycle. Adiponectin is reported to increase at weaning. Such higher levels are associated with elevated expression of the adiponectin receptor and apoptosis markers in epithelial cells, suggesting that this adipokine may play a role in the promotion of MG epithelial involution [21, 42].

The role of leptin and adiponectin contained in the milk is still under debate. Some evidences suggest that they may both promote intestine maturation in rodents and humans [42]. Mice born from mothers lacking or overexpressing adiponectin (AdipoQ-KO or AdipoQ-Tg, respectively) display growth retardation, alopecia and skin inflammation [45]. Furthermore, milk from AdipoQ-Tg mice is richer in lipid content, specifically in long-chain saturated fatty acids and ceramides, both types of lipids associated with inflammation. Different studies reported a negative correlation between milk leptin levels and infants’ weight [44]. On the other hand, in humans, elevated milk adiponectin levels were correlated with a slower growth trajectory during active breastfeeding, but greater growth at two years of age [46]. Nonetheless, investigations concerning leptin and adiponectin influence on infants feeding behavior are still conflicting and require further efforts [47].

MG adipocytes secrete IGF-1 which exerts a local action consisting of the promotion of the epithelial ductal growth [21]. Estrogen-induced expression of IGF-1 is more robust in MG adipocytes as compared to other adipose depots, supporting the idea that MG adipocytes may have unique properties compared to the ones present in other sites [21, 48]. MG adipocytes are also known to regulate angiogenesis within the gland through the production of VEGF, expressed in the mammary fat pad during the gland development and lactation [21]. HGF is secreted by human adipocytes and rodents preadipocytes and was demonstrated to regulate mammary epithelial morphogenesis [21, 49].

Importantly, fatty acids are also among the MG adipocytes secreted molecules potentially regulating epithelial growth. Different reports in fact support fatty acids ability promote ducto-alveolar development [21]. However, studies able to distinguish the differential effect of fatty acids secreted by MG adipocytes or the ones in circulation are needed in order to go in depth into the local and systemic effect of these molecules.

## Mammary gland adipocytes in obesity

Obesity is a disease characterized by an excessive accumulation of white adipose tissue, not only in fat depots but also ectopically, such that it increases the overall risk of patients’ morbidity and mortality [50]. It is recognized as a real pandemic whose prevalence in certain countries reaches 30–40% of the population and whose incidence is expected to increase during the next decades [50, 51]. According to the World Health Organization, obesity is defined by a body mass index (BMI) > or equal to 30 kg/m^2^ [52]. An important and understudied consequence of obesity is its poor breastfeeding outcome [28]. Women’s breastfeeding capabilities in fact reduce with increasing BMI [53], a phenomenon attributed to both, biologic and psychologic reasons [28]. Importantly, breastfeeding is considered as a protective factor against obesity development in children [54], and also against future abnormal glucose control in mothers [55]. Such interconnection triggers a vicious cycle according to which mothers who suffer from obesity perform poor breastfeeding abilities in turn increasing progeny’s risk of obesity onset [56]. Importantly, children with obesity are prone to maintain such disease throughout their lifespan, reason for which interventions targeting early risk factors are crucial [57, 58].

### Mammary gland development in obesity

As already described in Sect. 4, the genetic model of obesity ob/ob, does not develop a functional MG due to the lack of leptin action [25]. The leptin resistance typically documented in obesity [28] could thus contribute to the observed anomalies in lactogenesis. Importantly, leptin is also responsible for the normal functioning of the hypothalamic pituitary–gonadal axis. In fact, part of the phenotype described in leptin deficient mice is ascribed to the lack of a regular estradiol secretion which was proved to be necessary, but not sufficient, for the normal MG development [25]. A condition of hyperestrogenemia is typically observed in patients with obesity [59]. The aromatase enzyme, responsible for the conversion of androgens into estrogens, is in fact highly expressed and active in the adipose tissue of subjects with obesity, and is considered the responsible of the elevated estrogen levels in these patients [59]. Interestingly, according to some human studies, the condition of obesity-induced hyperestrogenemia could lead to adipocytes estrogen resistance [60, 61], possibly altering processes strictly dependent on estrogen action, such as MG development, remodeling and lactation. The potential effects of leptin and estrogen resistance on MG remodeling during the lactation cycle in condition of obesity still need to be explored.

**Fig. 6:**
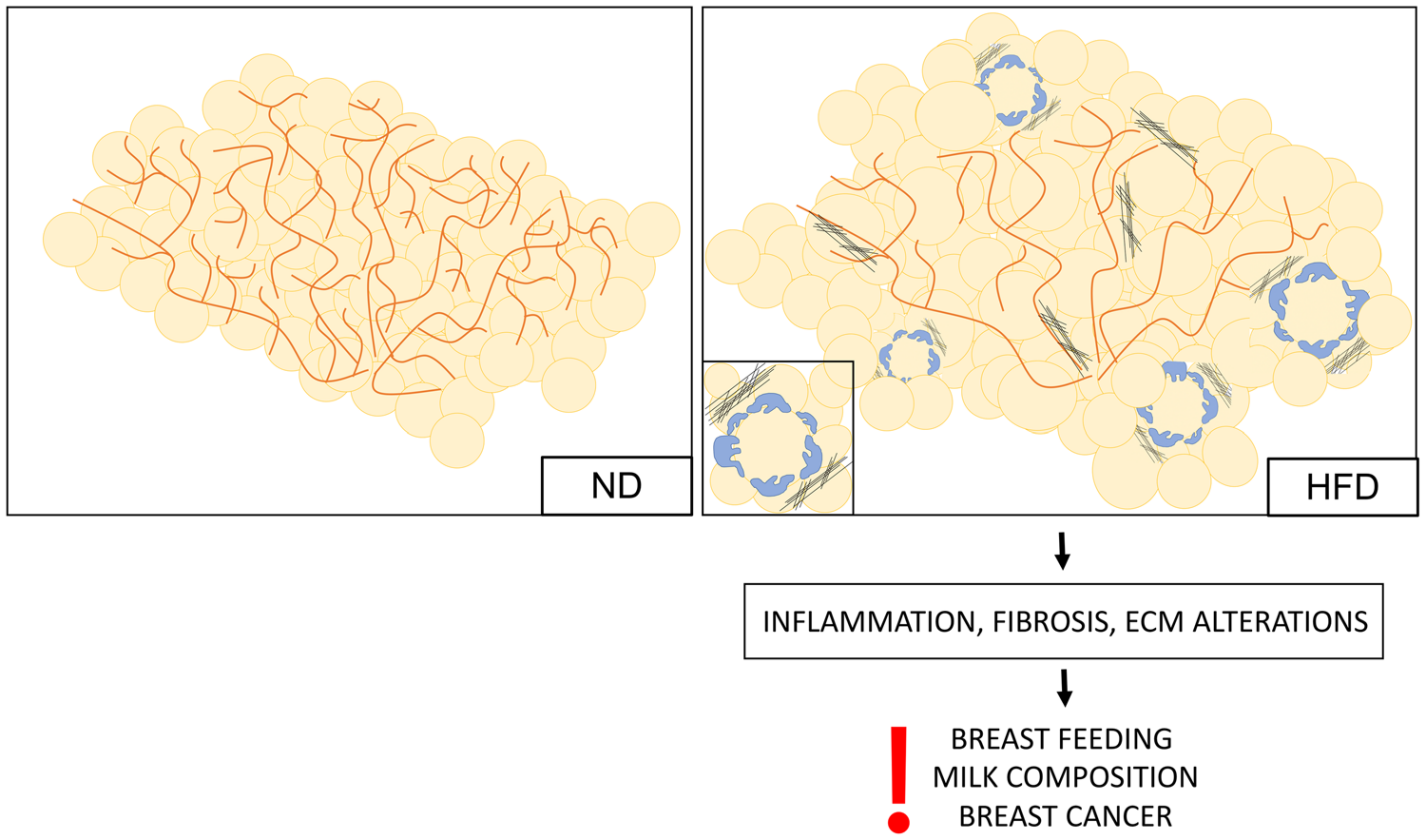
Scheme of mammary gland in condition of regular diet (ND) and high fat diet (HFD). The mammary gland in condition of HFD display a reduced number of ductal side branches, enlarged adipocytes and altered mammary stroma due to inflammation and fibrosis, as indicated by the presence of macrophages surrounding hypertrophic adipocytes and collagen fibers. These alterations compromise breastfeeding, alter milk composition and predispose to an increased risk for breast cancer development

**Fig. 7:**
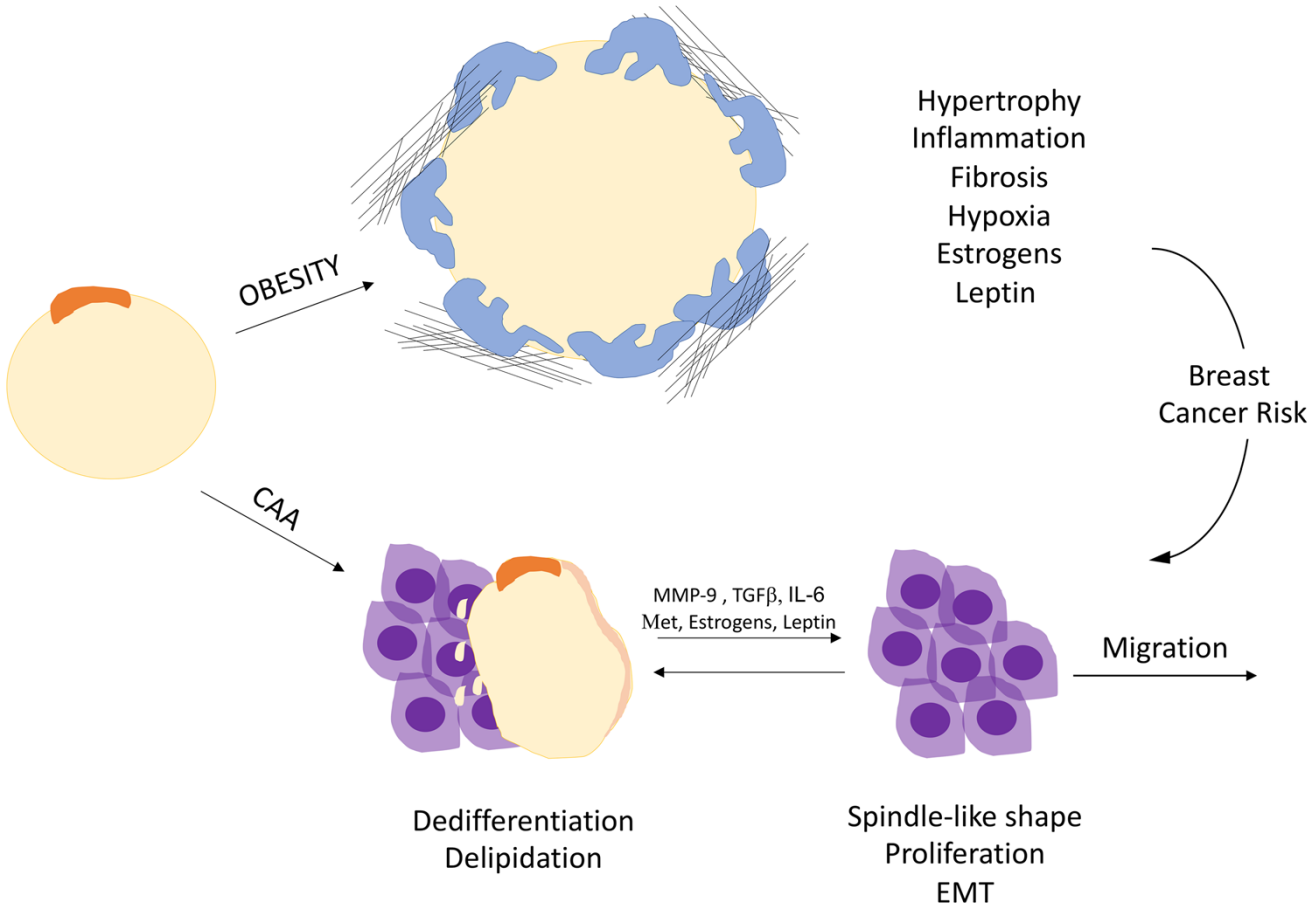
Scheme of mammary gland adipocytes in obesity and breast cancer. Obesity induces mammary gland adipocytes hypertrophy, thus fatty acid release, inflammation, fibrosis and hypoxia, accompanied by a state of hyperleptinemia and hyperestrogenemia, all features representing risk factors for breast cancer development. Cancer Associated Adipocyte (CAA) undergo delipidation and dedifferentiation upon the crosstalk with breast cancer cells. CAA release inflammatory cytokines, proteases, adipokines, estrogens and metabolites (Met) able to promote epithelio-mesenchymal transition (EMT), proliferation and migration of breast cancer cells

Consistently to what observed in genetic models, diet induced obese mice exhibit lower pregnancy success, higher stillbirth rate and impaired lactogenesis during the first post-natal days compared to regularly fed mice [31, 62]. The impaired lactogenesis was ascribed to the abnormal side-ductal branching and altered alveolar development at pregnancy day 14^th^ [31] and was associated with reduced pups’ body weight [31, 62] and increased mortality [62]. Kamikawa and colleagues revealed the presence of reduced ductal branching frequency and altered ductal layers due to incomplete myoepithelial lining in the MG of high fat diet fed mice as compared to normo-fed controls (Fig. 6) [63]. Furthermore, the abnormal MG morphology in virgin, pregnant and lactating models with obesity is characterized by the presence of adipocytes with a larger cross-sectional area [43, 63, 64]. Adipocytes hypertrophy is the *primum movens* of cellular death, macrophage infiltration and tissue inflammation (Fig. 6) [65, 66]. Cinti’s laboratory described the presence of macrophages forming “crown like structures” surrounding adipocytes debris and participating to the inflammatory-fibrotic process triggered by adipose cell death in both humans and mice [65–68]. Although such phenomenon is mainly observed in the visceral adipose depots [65–68], diet and genetic models of obesity (HFD and ob/ob) exhibit increased inflammation also in the MG [69]. These mice showed a higher presence of CLS, thus active macrophages surrounding debris of dead adipocytes, an elevation in the expression of the inflammatory markers *TNFα* and *IL-1β* and activation of the downstream regulator *NFkβ* [63, 69]. MG macrophages activation was demonstrated to be triggered by saturated fatty acids released by hypertrophic adipocytes (Fig. 6 and 7) [69]. Consistently, the number of MG’s CLS was correlated with adipocytes area and BMI in women with breast cancer [70]. In addition, since obesity is associated with reduced adiponectin expression and adiponectin deficient mice display increased MG inflammation, it is possible that the obesity induced MG inflammation is also mediated by the lower levels of this adipokine [45]. Importantly, obesity and adipocytes hypertrophy are also associated with tissue fibrosis [28, 63, 71–73]. MG remodeling during the lactation cycle involves a deep modification of the extracellular matrix (ECM) which is strictly required to allow the proper gland development and functioning [28]. Consistently, Kamikawa’s group reported increased collagen layers surrounding the ductal structures of high fat-fed mice compared to normal-fed controls [63]. Based on these evidences, adipocytes hypertrophy, thus inflammation, fibrosis and death, and the consequent alteration in the mammary microenvironment, may compromise the proper MG remodeling and functioning and explain the poor lactation outcomes observed in condition of obesity. However, the exact mechanism at the basis of such phenomenon are poorly investigated even though it clearly holds critical clinical implications not only for the comprehension of obesity related poor breastfeeding, but also in the context of breast cancer research, as discussed in Sect. 7.

### Milk production in obesity

The described impaired lactogenesis in animal models of diet induced obesity is also associated with alteration in milk yield and composition [31, 43, 64]. According to one study, upon delivery, obese mice presented an increased accumulation of lipids in the epithelial cells which was associated with poor milk excretion [31]. The delay in lactogenesis was accompanied by a reduction in the expression of the milk proteins *WAP*, *β-casein* and *α-lactalbumin* and by a decrease in *de novo* lipid syntheses (reduced acetyl-CoA carboxylase content in both adipocytes and epithelial cells) compared to chow or low-fat fed animals [31, 64]. Abnormalities in alveolar development and milk composition (except for *α -lactalbumin*) were normalized by day 10 of lactation [31], although abnormal lipid quality, consisting of a low presence of medium chain fatty acids and high prevalence of long chain fatty acids, was reported thorough the whole lactation period by different investigations [62, 64]. Importantly, since *α -lactalbumin* mediates lactose synthesis, which is the osmotic drive for milk excretion, pups of obese mothers may be nourished with a lower milk volume [31, 43]. In fact, milk total yield and contents of water, omega-3 and -6 fatty acids were reported to be lower in obese rats compared to controls, whereas total fat, amminoacids and leptin contents were recorded as higher [43]. Nevertheless, in the study of milk composition it is critical to isolate the individual effects of diet and obesity. One study observed reduced milk fat content and altered lipid composition dependent on obesity and high fat diet, respectively [64]. Furthermore, the same study reported reduced epithelial Plin2 content (protein coated to lipid vacuoles and regulating their release) in obese lactating mice [64].

These *in vivo* findings are coherent with the impaired lactogenesis observed in women suffering from obesity who also display early breastfeeding cessation [53, 74]. However, further studies are required to explore in depth such mechanism in humans with obesity, especially the role of dysfunctional adipocytes on MG development and lactation cycle remodeling.

## Mammary gland adipocytes in breast cancer

Since adipocytes are the most prevalent cell type of the mammary stroma, lots of recent efforts have been made to understand their role in the initiation and progression of breast cancer. Several reports have therefore emphasized dysfunctional adipocytes ability to influence tumor initiation, growth and metastasis [75–77]. For many cancer types in fact, the magnitude of adipose tissue invasion mirrors tumor aggressiveness and consequently reflects a poor prognosis [76–78]. Consistently, breast tumor progression and metastasis prevail in adipocytes rich microenvironments [77, 78]. Adipocytes directly participate to tumor growth through their endocrine/paracrine activity and remodeling of tissue microenvironment [72, 75–77, 79, 80]. The secretion of growth factors, inflammatory cytokines, adipokines, estrogens and metabolites, together with the production of ECM components, can deeply influence tumor initiation, metabolism and expansion [75, 77]. Furthermore, breast cancer cells directly crosstalk with adjacent adipocytes which in turn undergo a phenotypic transition able to support tumor growth and metastasis, becoming what have been defined as *Cancer Associated Adipocytes* (CAA) [81]. CAA are considered as an intermediate state of *Cancer Associated Fibroblasts*, deeply investigated for their ability to support tumor growth [28, 82]. Importantly, adipocytes may also deeply impact cancer therapeutic success due to their ability to sequester and metabolize chemotherapeutics (e.g., daunorubicin) [83]. The mechanism at the basis of the association between breast cancer, obesity and adipocytes dysfunction are therefore multiple and will be briefly addressed in the following sections.

### Breast cancer associated adipocytes

Co-culture experiments of mature adipocytes and breast cancer cells revealed the existence of an intense crosstalk between these two cell types [81, 84]. Cancer cells, in fact, deeply modified adipocytes gene expression and secretory profile, while increasing their own invasive abilities [81, 84]. Specifically, adipocytes changes included delipidation, dedifferentiation and increased expression of inflammatory cytokines (e.g. IL-6, IL1β) and proteases, in both human and murine cell culture [81]. Furthermore, a recent study also suggested CAA transdifferentiation toward a brown phenotype [85]. MG human biopsies also showed intense positivity for IL-6 and the metalloprotease MMP-11 in adipocytes adjacent to invading cancer cells as compared to the distant ones, which in turn displayed higher adiponectin expression and larger cross-sectional area [81]. Hence, breast cancer cells induce adipocytes IL-6 secretion, which is in turn responsible of the higher breast cancer aggressiveness [81]. This study underlined the characteristics of CAA: adipocytes adjacent to the tumor, displaying a well-defined phenotype induced by cancer cells and able to promote tumor expansion and invasion (Fig. 7) [81, 84]. In this experimental system, breast cancer cells acquire a spindle-like shape, lose their epithelial polarity and undergo an epithelial-mesenchymal transition (EMT) responsible for cellular dispersion and increased mobility [84]. This process is mediated by different adipocytes secreted molecules such as MMP-9 [84] and TGFβ [86, 87]. In addition, according to a recent study, leptin disrupts epithelial polarity and promotes premalignant alteration in the mammary gland, a finding that further links obesity hyperleptinemia with breast cancer risk [88]. Importantly, not all breast cancer cell lines undergo proliferation upon co-culture with adipocytes, while the effect on cell migration seem to be common to different breast tumor subtypes [81, 84]. Likewise, such outcomes are observed only in co-cultures settings including mature adipocytes as opposed to preadipocytes [81]. Interestingly, a recent study emphasized the possibility to induce the differentiation of breast cancer cells which underwent EMT into mature post-mitotic adipocytes through the combined use of MEK inhibitors and the antidiabetic drug Rosiglitazone, which exerts an adipogenic effect [87]. Such experiment was successfully performed on different murine and human models of breast cancer and provoked the repression of primary tumor invasion and metastasis, an evidence that emphasizes the potential exploitation of EMT for therapeutic purposes [87]. Another crucial aspect concerning adipocytes-breast cancer cells interaction is related to the energy demand: breast cancer cell can undergo a metabolic switch to better adapt to different microenvironments and are able to promote adipocytes metabolic reprogramming in order to support their energy supply. According to co-culture experiments in fact, adipocytes derived free fatty acids were transferred to breast cancer cells supporting their proliferation and migration [89]. The same phenomenon was further enhanced when co-culturing obese adipocytes [89]. Breast cancer cells can thus exploit the neighboring adipocytes to acquire energy-dense substrates to burn either via glycolytic or oxidative phosphorylation pathways. Other adipocytes secreted metabolites such as β-hydroxybutyrate, pyruvate and lactate are also able to support breast tumorigenesis [90, 91].

### Dysfunctional adipocytes linking obesity and breast cancer

Obesity is widely accepted as a risk factor for the development of breast cancer [59, 77]. Being one of the most common type of tumor in women, mammary neoplasia caused over 41,000 death in the United States in 2019 [92]. The modified secretome of CAA shares several features in common with hypertrophic dysfunctional adipocytes observed in condition of obesity (Fig. 7). They in fact release: inflammatory cytokines such as TNFα, IL-1β, IL-6; chemokines (e.g. CCL2 and CCL5) attracting M1 proinflammatory macrophages; abnormal ECM components likewise contributing to microenvironment anomalies commonly observed in breast tumors; fatty acids, the tumor energetic substrate, which also foster the inflammatory process [28, 75]. The chronic inflammatory status observed in patients with obesity is hence considered a risk factor for the development of breast cancer and is correlated with interstitial fibrosis, also involved in the expansion of the neoplastic lesion [70, 79, 93]. As previously described, the number of MG’s CLS was in fact correlated with adipocytes area and BMI in women with breast cancer [70]. The obesity induced interstitial fibrosis favors breast carcinogenesis by modifying ECM mechanics [93]. Specifically, according to one study, the obese MG fat pad contains more myofibroblasts which produce more fibrillar and stiffer ECM, which increase the malignant behavior of breast cancer cells [93]. Furthermore, in condition of obesity, dysfunctional adipocytes produce high levels of collagen [71, 80], in turn responsible for hypoxia, inflammation [72] and for the promotion of a breast protumorigenic environment [79]. Adipocytes released endotrophin, a product of collagen VI cleavage, was demonstrated to promote fibrosis, angiogenesis and inflammation and was associated with aggressive mammary tumor growth in a process partially mediated by TGFβ [80]. The inflammatory storm is also responsible for the induction of the aromatase enzyme expression in the MG of obese models [69]. Inflammatory cytokines not only increase with obesity and breast cancer, but also with menopause. Adipocytes are the main source of estrogens after menopause and, as described in the previous section, the increased aromatase activity and expression in the expanded adipose tissue are at the basis of the state of hyperestrogenemia accompanying obesity and linking such disease to the elevated risk for breast cancer development [59]. According to a recent study, estrone dominates after menopause and, differently from 17β−estradiol, it exerts a pro-inflammatory effect stimulating NFkβ-induced cytokine release [94]. Such process is observed in the MG upon adipocyte-cancer cell interaction and support tumor initiating stem cells and estrogen receptor + (ER +) cancer growth [94]. For these reasons, an elevated estrone/17β-estradiol ratio is an indicator of poor prognosis and inhibition of the enzyme converting 17β-estradiol into estrone holds critical therapeutic potentials [94]. Additional MG adipocytes secreted molecules such as autotaxin, resistin, IGF-1, HGF, VEGF participate to breast tumorigenesis and expansion and are abnormally regulated in condition of obesity. For a deeper review of the topic please refer to the following articles [76, 77, 79, 91]. The intricated vicious cycle orchestrated by cancer cells and dysfunctional adipocytes emphasize the complexity of the mechanism linking obesity and breast cancer. The multifaceted role played by MG adipocytes in these conditions holds critical clinical implications and must be further investigated.

Another aspect worth briefly mentioning concerning adipocyte and breast cancer is related to MG remodeling during the lactation cycle. MG involution and tumorigenesis have in fact some features in common, such as inflammation, fibrosis and apoptosis [3, 95–97] which predispose to the so called pregnancy-associated breast cancer (PABC) development [95]. PABC diagnosed within 2 years post-partum [95], occurs in women < 45 years old and confers a threefold higher risk of metastasis and death [98]. Consistently, abnormal post-lactational involution is associated with a higher relative risk of PABC development [96]. High BMI is frequent among women with this specific subtype [95]. Although, a bidirectional interplay between breast cancer risk and MG’s adipocytes has been described, the mechanisms involved have not been completely elucidated and most of the studies on obese MG adipocytes is focused on aspects other than the lactation cycle [3].

## Conclusions and future perspectives

The described evidences underline the critical role of MG adipocytes in several physiologic and pathologic processes. During the last few years efforts have been made to investigate cancer associated adipocytes function, however additional studies are needed to explore MG adipocytes role during the lactation cycle, their impact on neonates’ health and how such phenomena are influenced by obesity. Thanks to the recent advances in technology, only during the last couple of years multiple adipocytes types with different specific functions have been identified in distinct adipose depots [99–101]. It is thus possible that the MG is composed by adipocytes subpopulations with different specialized functions as compared to adipocytes belonging to other fat depots. Further efforts are needed to deconvolute the complex and multiple functions of the adipose organ in all its different depots, including the MG. Uncovering the dynamic displayed by MG adipocytes during the lactation cycle in physiologic condition and in the context of obesity will have implication of great clinical relevance for the study of the poor breast feeding outcomes observed in obesity and breast cancer cell-adipocytes interactions [3, 102].

## Abbreviations

MG: Mammary gland
WAT: White adipose tissue
BAT: Brown adipose tissue
LD: Lypodistrophy
HFD: High fat diet
ob/ob: Genetic mouse model lacking leptin
db/db: Genetic mouse model lacking leptin receptor
